# MicroRNA-153 attenuates hypoxia-induced excessive proliferation and migration of pulmonary arterial smooth muscle cells by targeting ROCK1 and NFATc3

**DOI:** 10.3892/mmr.2022.12848

**Published:** 2022-09-08

**Authors:** Minjie Zhao, Wei Wang, Ya Lu, Nan Wang, Delei Kong, Lina Shan

Mol Med Rep 23: 194, 2021; DOI: 10.3892/mmr.2021.11833

Subsequently to the publication of the above paper, the authors have realized that the image chosen to represent the ‘Mimic control’ experiment in Fig. 4G was inadvertently selected incorrectly; the data originated from the same source as that chosen (correctly) for the ‘Inhibitor control’ experiment in the same figure.

The revised version of Fig. 4, now containing the correct data for the ‘Mimic control’ experiment in Fig. 4G, is shown below. Note that this error did not quantitatively affect either the results or the overall conclusions of this study. All the authors agree with the publication of this corrigendum, and are grateful to the Editor of *Molecular Medicine Reports* for allowing them the opportunity to publish this. They also wish to apologize to the readership of the Journal for any inconvenience caused.

## Figures and Tables

**Figure 4.**
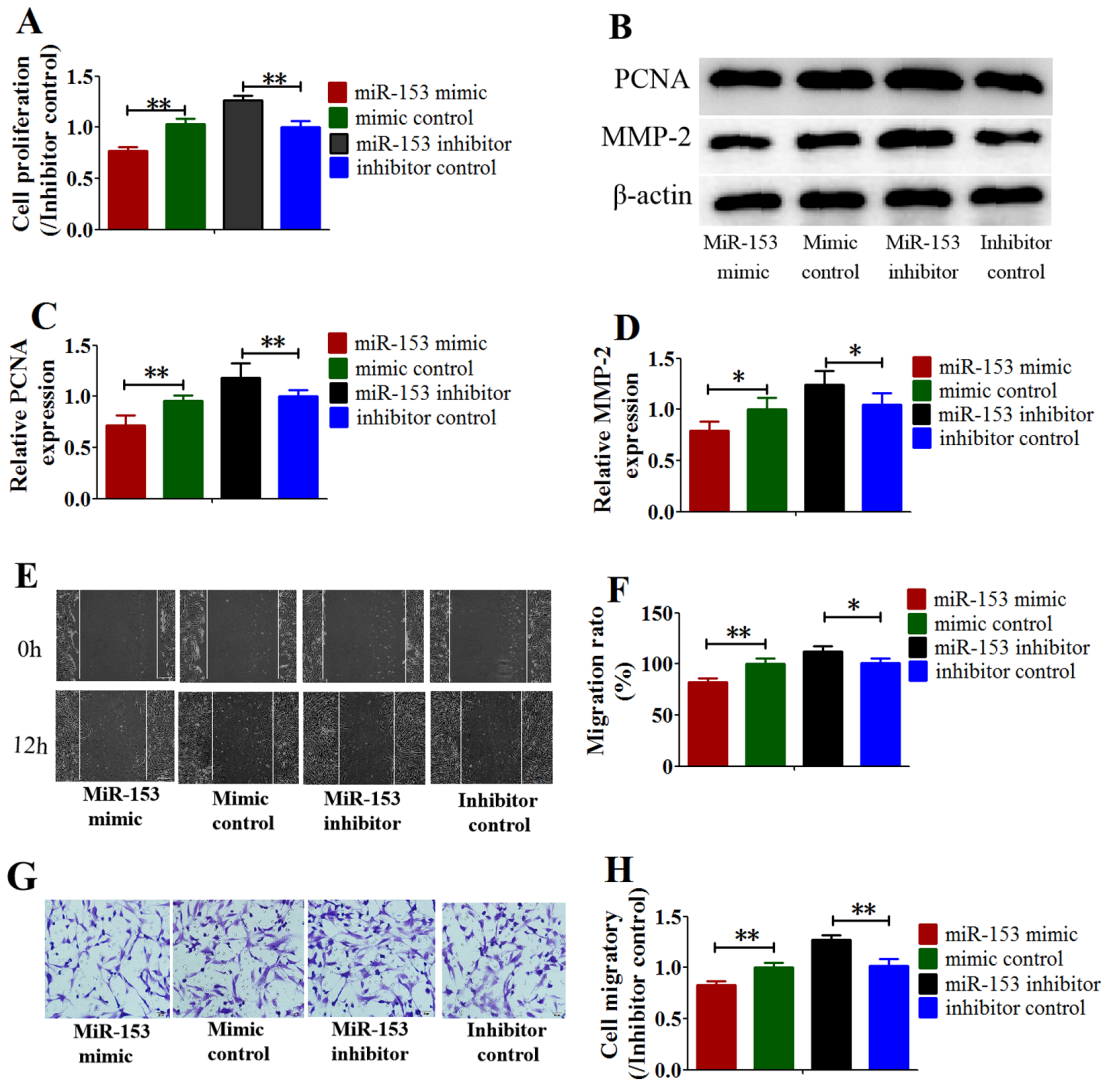
miR-153 inhibited hypoxia-induced proliferation and migration capacity of HPASMCs. (A) CCK-8 and (B-D) western blot assays analyzed the cell proliferation; (E and F) wound healing. Scale bar, 200 µm. (G and H) Transwell assays for cell migration. Scale bar, 50 µm. Data represent the mean ± standard deviation. n=3. *P<0.05, **P<0.01 vs. inhibitor control. miR, microRNA; HPASMCs, human pulmonary artery smooth muscle cells.

